# Supplementary material for: Chromothripsis during telomere crisis is independent of NHEJ, and consistent with a replicative origin
Source: Genome Res. 2019 May;29(5):737–49. doi: 10.1101/gr.240705.118 (PMC6499312; doi:10.1101/gr.240705.118)
Supplement: Supplemental Material [file supp_gr.240705.118_Supplemental_file_1.zip › contigs/annotated_contigs/DB77/contig.3.DB77_length_309_mean_cov_4.60517799353.docx]

**DB77_length_309_mean_cov_4.60517799353**

```
ACTGCACCCAGCCTCTAATTCTAATTTATTAATTGAATAAATTCTCTTCATTAACTGACATATAAAACAACACATTTGTCAGATAAATG
 >chr9:30560801-30560929 + E=1e-65
ACCTTATATCAGGTACTTAGGCAAAATATCATGTTCTT|T|TTTTTTTTTTTTTTGAGACGGAGTCTCGCTCTGTCGCCCAGGCCGGAC
                                       >chr9:39599625-39599683 + E=2e-20
TGCGGA|CTT|TAGAGTAGTTGTGATTTGGATTGCCAGTTGTTTGAGAAGTCATCTTTTTTTAATCTATCTGGTTAGAAGAAGATCTGG
       >chr9:30560925-30561052 + E=4e-65
TAGATAGACAGAACCAGAAGCCTCACTGGTTAACTACATGCAACTA
```
